# ASSESSING THE IMPACT OF THE PATIENT-DRIVEN PAYMENT MODEL ON NURSING HOME CARE QUALITY: A STAFFING PERSPECTIVE

**DOI:** 10.1093/geroni/igae098.1386

**Published:** 2024-12-31

**Authors:** Gregory Orewa, Robert Weech-Maldonado, Ganisher Davlyatov, Justin Lord, David Becker, Sue Feldman

**Affiliations:** The University of Texas San Antonio, San Antonio, Texas, United States; University of Alabama at Birmingham, Birmingham, Alabama, United States; University of Oklahoma Health Sciences Center, Oklahoma City, Oklahoma, United States; Louisiana State University Shreveport, Shreveport, Louisiana, United States; University of Alabama at Birmingham, Birmingham, Alabama, United States; University of Alabama at Birmingham, Birmingham, Alabama, United States

## Abstract

The Patient-Driven Payment Model (PDPM) launched on October 1, 2019, restructured Medicare payments to reflect resident needs over service quantity, aiming to improve quality and efficiency in nursing homes (NH). PDPM’s shift towards resident needs (rather than therapy minutes) may lead to greater nurse utilization, which may potentially improve the quality of care. This study draws on the resource-based view (RBV) theory to understand the association between the PDPM and NH quality of care mediated by nurse staffing intensity. Utilizing a longitudinal design (2017-2021), seven different datasets were merged (Cost reports, Payroll Base Journal (PBJ), Care Compare, Area Health Resource File (AHRF), HHS Provider Relief Fund, CDC NH COVID-19 public files, and CDC COVID-19 Data Tracker). Structural equation modeling (SEM) was used to assess the direct and indirect effects of PDPM on care quality, with nursing staffing intensity serving as a mediator. NH quality of care was operationalized using quality-measure five-star rating and staffing intensity by RN, LPN, and CNA hours per resident. We examined the direct effect of PDPM on quality of care and the indirect effect of PDPM on quality through nurse staffing. Control variables included organizational and market-level factors, and COVID-19-related variables. The result suggests an increase in RN staffing post-PDPM but a decrease in CNA hours (p< 0.001). PDPM had an indirect positive effect on quality through RNs while negative through CNAs. PDPM also showed a positive direct effect on quality. Policy implication highlights the complex interplay between different staffing roles and the impact on quality.

